# SDHB Suppresses the Tumorigenesis and Development of ccRCC by Inhibiting Glycolysis

**DOI:** 10.3389/fonc.2021.639408

**Published:** 2021-05-19

**Authors:** Zhiyu Fang, Qiang Sun, Huihui Yang, Junfang Zheng

**Affiliations:** ^1^Beijing Key Laboratory of Cancer Invasion and Metastasis Research, Department of Biochemistry and Molecular Biology, School of Basic Medical Sciences, Capital Medical University, Beijing, China; ^2^Blood Purification Center, Beijing Key Laboratory of Pediatric Chronic Kidney Diseases and Blood Purification, Key Laboratory of Major Diseases in Children, Ministry of Education, Beijing Children’s Hospital, Capital Medical University, National Center for Children’s Health, Beijing, China

**Keywords:** SDHB, renal cell carcinoma, prognosis, methylation, glycolysis

## Abstract

Metabolic reprogramming is the prominent feature of clear cell renal cell carcinoma (ccRCC). Succinate dehydrogenase subunit B (SDHB) is one of subunits of mitochondrial respiratory chain complex II. The loss of SDHB function is closely related with metabolic changes in kidney cancer cells. However, the role and molecular mechanism of SDHB in ccRCC occurrence and progression are still unclear. In this study, the results of bioinformatics analyses on GEO, TCGA and oncomine databases and immunohistochemistry showed that the expression level of SDHB was downregulated in ccRCC tissues. SDHB level was gradually downregulated as ccRCC stage and grade progressed. The low level of SDHB was associated with poor prognosis of ccRCC patients, especially for advanced ccRCC patients. Increased methylation levels in *SDHB* gene promoter led to the downregulation of SDHB level in ccRCC tissues. SDHB was correlated with many metabolism related genes and its interacting proteins were enriched in metabolic pathways. SDHB overexpression suppressed the proliferation, colony formation and migration of ccRCC cells by inhibiting aerobic glycolysis. SDHB may be a potential prognostic marker and therapeutic target for ccRCC.

## Introduction

Kidney cancer accounts for 2-3% of all adult malignant tumors, and its incidence ranks sixth among men and tenth among women (1). Renal cell carcinoma accounts for 90% of all kidney tumors (2). The traditional morphological classification of these tumors divides them into three main subtypes: clear cell, papillary, and chromophobe subtypes (3). Clear cell renal cell carcinoma (ccRCC) is the most common histological subtype (80% to 90%) (4). Although the molecular targeted therapy of ccRCC has made great progress, the therapeutic effect is not yet satisfactory (5). The molecular mechanism of ccRCC tumorigenesis and development helps to identify the novel therapeutic targets of ccRCC. However, its molecular mechanism has not yet been fully clarified (6). Metabolic reprogramming can lead to tumorigenesis (7–9). In ccRCC, prominent changes in metabolism occur, and ccRCC is described as a “cell metabolic disease”. Abnormal glucose metabolism, i.e. increased glycolysis, even aerobic glycolysis (also called Warburg effect), is very obvious in ccRCC (10–13). Therefore, the abnormal metabolic pathways may be potential targets for more effective ccRCC treatment (14).

Succinate dehydrogenase (SDH, also known as mitochondrial respiratory chain complex II) is a key respiratory enzyme located on the inner mitochondrial membrane, which links the tricarboxylic acid (TCA) cycle with oxidative phosphorylation and plays the important roles in both TCA cycle and oxidative phosphorylation (15). SDH consists of four nuclear coding subunits (SDHA/B/C/D). SDHA and SDHB are catalytic subunits, SDHC and SDHD provide binding sites for ubiquinone (an element of the electron transport chain) (16). The low expression of SDHB promotes aerobic glycolysis (17, 18), and the lack of SDHB function leads to the occurrence and development of multiple kinds of tumors, including liver cancer and colorectal cancer *etc*. (18, 19). In kidney cells of *sdhb* knockout mice, the TCA cycle is completely blocked, and the Warburg effect is enhanced (20). In ccRCC cells, the Warburg effect is more pronounced (21). The loss of SDHB function is closely related with metabolic changes in kidney cancer cells (22, 23). However, the role of SDHB in ccRCC and whether it affects ccRCC by regulating the level of aerobic glycolysis remain unknown.

This study proved that SDHB expression level was downregulated in ccRCC tissues. The increased methylation level in *SDHB* gene promoter led to the downregulation of SDHB level. SDHB overexpression suppressed ccRCC cell proliferation, colony formation and migration *in vitro* by inhibiting glycolysis.

## Materials and Methods

### Bioinformatics Analyses

The microarray series (GSE53757) information containing ccRCC tumors and matched normal samples was from the National Center for Biotechnology Information Gene Expression Omnibus database (NCBI GEO, https://www.ncbi.nlm.nih.gov/gds/?term=GSE53757). The Cancer Genome Atlas kidney cancer database (TCGA_KIRC) mRNA data (RNA Seq v2) and the clinical data of ccRCC patients (TCGA, Nature 2013) were from https://www.synapse.org/ and the cBioPortal database (https://www.cbioportal.org/), respectively. Oncomine renal cancer data for SDHB between tumor and non-tumor samples (https://www.oncomine.org/) were selected with a threshold of *P* value ≤ 1E-4, fold change ≥ 2 and top 10% gene rank. The protein levels of SDHB were downloaded from the UALCAN database (http://ualcan.path.uab.edu/).

### Tissue Collection

Primary ccRCC and matched adjacent normal kidney tissues from the same patient (n=75) were obtained from nephrectomy specimens at the Affiliated Beijing Friendship Hospital, Capital Medical University from April 2018 to March 2019. Specimens were collected immediately after nephrectomy, formalin-fixed and paraffin-embedded for use in immunohistochemistry (IHC) analysis. All specimens were histologically confirmed by pathologists. The study was approved by the Research Ethics Board of Affiliated Beijing Friendship Hospital and was performed according to the World Medical Association Declaration of Helsinki. All subjects included in the protocol signed a declaration of informed consent. Prior to surgery, the patients had not received chemotherapy or radiotherapy.

### Immunohistochemistry

Immunohistochemistry (IHC) was performed as previously reported (24). The sections were incubated with rabbit anti-SDHB monoclonal antibody (Abcam, Cambridge, UK, Cat#ab175225, 1:100) under optimal conditions. The optical density was analyzed using Image-Pro plus 6.0 (Media Cybernetics Inc. Silver Spring, MD).

### Gene Set Enrichment Analysis

By calculating the pathway enrichment score (ES), gene set enrichment analysis (GSEA) was performed according to the previous method (25) to evaluate whether genes from a predefined gene set were enriched in the highest/lowest rank genes.

### Analyses About Methylation Level of SDHB Gene Promoter and Its Correlation With Phenotypes

The methylation levels of *SDHB* gene promoter were from the UALCAN database. The correlation between the methylation levels of *SDHB* gene promoter and clinical phenotype was analyzed from the MEXPRESS database (https://mexpress.be/) (26).

### Plasmid Construction, Cell Culture, Transfection and Treatment

SDHB overexpressing plasmid was constructed by amplifying the corresponding sequences and ligation into pcDNA3.1-flag vector. Sequences were verified by PCR amplification. The human renal carcinoma cell line ACHN and ccRCC cell line 786-O were obtained from American Type Culture Collection (ATCC, Manassas, VA, USA). Cells were grown in RPMI-1640 medium containing 10% fetal bovine serum and 1% streptomycin/penicillin, at 37°C and 5% CO_2_. All cell culture reagents were provided by HyClone (Logan, UT, USA). Cells were transfected by using Lipofectamine 2000 (Invitrogen, Carlsbad, CA, USA), and were pretreated with DNA demethylating drug decitabine (Selleck, Cat#S1200) at different concentrations for 12 h.

### Western Blotting

Western blotting (WB) was performed as previously described (25). Anti-SDHB and anti-β-actin antibodies were purchased from Abcam (Cat#ab175225) and Sigma–Aldrich (St. Louis, MO, Cat#A1978), respectively.

### Bisulphite Modification and Methylation-Specific PCR (MSP)

Genomic DNA extraction kit (DC102, Vazyme) was used to extract genomic DNA of kidney cancer cells, and bisulfite conversion kit (EM101, Vazyme) was used to denature DNA and convert with bisulfite. Methylation-specific PCR was performed using specific primers designed to amplify methylated and unmethylated putative SDHB promoter sequences (GeneBank accession No. U17296): unmethylated–specific, 5′-TGTGTTGTTATTGTGTTATTGTGTAT-3′ (forward) and 5′-CCACCAAAAATTATAACCAACAACCA-3′ (reverse) and methylated–specific, 5′-TGCGTCGTTATTGCGTTATTGCGTAC-3′ (forward) and 5′-CCGCCAAAAATTATAACCGACAACCG-3′ (reverse). Methylation-specific PCR kit (EM201, Vazyme) for methylation-specific PCR was used.

### CCK-8 Assay

Cells were seeded in 96-well plates (3000 cells per well). Plates were then incubated for 24-96 h, and viable cells were analyzed with Cell Counting Kit-8 (Dojindo, Kumamoto, Japan) by using a Enspire, microplate reader (Perkin Elmer, Waltham, MA, USA) at 450 nm.

### Colony Formation Assay

The ability of single cells to form colonies in a six-well plate was used to determine the survival of ACHN and 786-O cells stably expressing SDHB (500 cells/well) for 12 days. The colonies were fixed and stained with 0.05% crystal violet (Beyotime, China) before counting. Triplicate experiments with triplicate samples were performed.

### Wound Healing Assay

The cell migration was evaluated by the wound healing test. The cells were plated in a six-well culture dish, and the cell monolayer was scraped off with the tip of a P-20 microtube. Wound healing was monitored and measured.

### Co-Expression Gene Network of SDHB

Co-expression online analysis was performed in the website (https://www.cbioportal.org/) by using the mRNA level in the TCGA_KIRC database (TCGA, Nature 2013). With *P* value < 0.05 as the threshold, the genes which had greater than 0.4 Spearman correlation coefficient with SDHB in expression level were uploaded to Cytoscape software (Cytoscape_v3.8.0) to draw gene co-expression network.

### Protein-Protein Interaction (PPI) Network Construction of SDHB-Related Genes

GEO2R was used to compare the mRNA expression levels of ccRCC tumors and matched normal samples from the GSE53757 dataset, and differentially expressed genes (DEGs) was screened. The cutoff criteria were *P* value < 0.05 and |logFC| > 1. The DEGs which were statistically correlated with SDHB (|spearman coefficients| > 0.4) were defined as SDHB-related genes.

Then STRING (https://string-db.org/), an online biological database, was used to predict the interaction among SDHB-related DEGs. The minimum interaction value is set to 0.4 (medium confidence), and protein nodes that have not interacted with other proteins are removed. Then the PPI data were uploaded into Cytoscape software to construct a PPI network.

### GO and KEGG Pathway Analyses

To explore the functional annotation and involved pathways of SDHB-related DEGs, the GO and the KEGG pathway enrichment analyses were executed by online analysis tools–Database for Annotation, Visualization, and Integrated Discovery (DAVID) (https://david.ncifcrf.gov/) against the background of *Homo sapiens*.

### Determination of Metabolites Concentration

Glucose concentration in culture medium and intracellular lactate concentration were determined using glucose concentration assay kit (BC2500, Solarbio) and lactic acid assay kit (BC2235, Solarbio), respectively. All these assays were performed according to the manufacturer’s protocol.

### Statistics

All experiments were repeated three times. The results of paired samples were analyzed using paired samples *t*-test. The independent samples *t*-test was used to analyze the statistical significance of unpaired samples. The samples more than two groups were analyzed with analysis of variance (ANOVA). The log-rank test for the generated Kaplan–Meier (KM) curve was conducted to evaluate the association between the expression level of SDHB and the survival rate. Proliferation curve result was analyzed by repeated measures ANOVA. Correlation between genes expression levels was analyzed by Spearman correlation analysis. Statistical significance was set at two-tailed *P* values < 0.05. All statistical analyses were performed using SPSS Statistics 19.0 (IBM SPSS, Chicago, IL) and Graphpad Prism 5 (Graphpad Inc., San Diego, CA).

## Results

### SDHB Expression Level Is Downregulated in ccRCC Tissues

**Figure 1:**
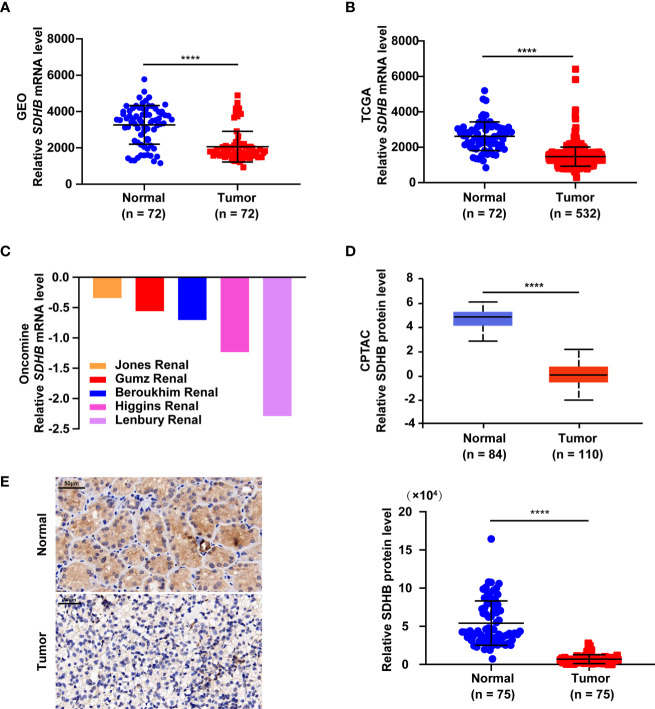
SDHB expression level is downregulated in ccRCC tissues. **(A–C)** Comparison of *SDHB* mRNA levels between adjacent normal tissues and ccRCC tissues based on GEO GSE53757 data **(A)**, TCGA_KIRC data **(B)**, and oncomine data **(C)**, respectively. Oncomine data included Jones Renal (normal = 23; ccRCC = 32), Higgins Renal (normal = 3; ccRCC = 36), Beroukhim Renal (normal = 11; ccRCC = 32), Gumz Renal (normal = 10; ccRCC = 10), Lenbury kidney (normal = 9; ccRCC = 9). **(D)** Comparison of SDHB protein levels between adjacent normal tissues and ccRCC tissues based on CPTAC data. **(E)** Representative IHC figure of SDHB expression and scatter plot displaying the expression of SDHB in adjacent normal tissues and ccRCC tissues. *P* value was derived from independent sample two tailed *t*-test. The data were presented as mean ± SD. ****P < 0.0001.

In order to clarify the role of SDHB in ccRCC, we first analyzed the expression levels of SDHB from multiple databases. Data from GEO database (GSE53757) and the TCGA_KIRC database suggested that *SDHB* mRNA levels were significantly downregulated in ccRCC tissues (**Figures 1A, B**). Five datasets in the Oncomine database also revealed that compared with normal tissues, *SDHB* mRNA levels in ccRCC tissues were significantly reduced (**Figure 1C**). The results from CPTAC database further showed the protein levels of SDHB in ccRCC tissues were lower than those in adjacent normal tissues (**Figure 1D**). Subsequently, we detected SDHB protein levels in newly collected ccRCC tissues by IHC and results further confirmed that the SDHB protein levels were significantly downregulated (**Figure 1E** and **Supplementary Table 1**).

### SDHB Is a Potential Prognostic Marker for ccRCC Patients

**Figure 2:**
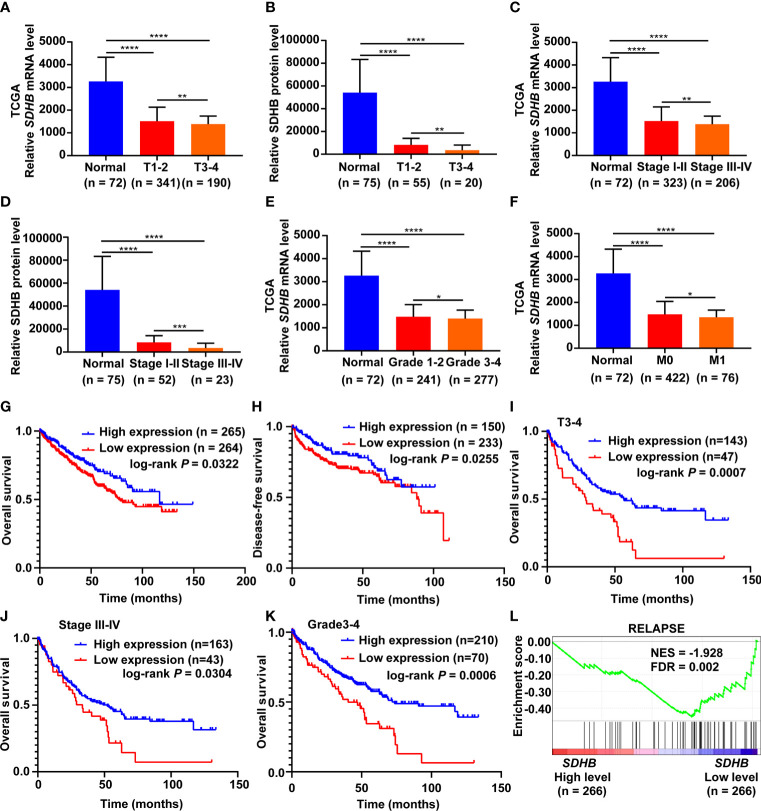
SDHB is a potential prognostic marker for ccRCC patients. **(A–E)** SDHB mRNA and protein levels were gradually downregulated as T stage, AJCC stage and Fuhrman grade progressed. **(F)** *SDHB* mRNA level was negatively correlated with distant metastasis of ccRCC patients. **(G, H)** The Kaplan-Meier (KM) curves of overall survival and disease-free survival based on TCGA_KIRC data. ccRCC patients were divided into high/low expression groups according to *SDHB* mRNA level. **(I–K)** KM curves of overall survival based on TCGA_KIRC data. Advanced ccRCC patients were divided into high/low expression groups according to *SDHB* mRNA level. **(L)** Enrichment plot of gene expression signature for relapse (KAUFFMANN_MELANOMA_RELAPSE_UP) was obtained by GSEA according to *SDHB* mRNA levels. The ccRCC samples from TCGA_KIRC database were divided into high and low SDHB expression groups according to the median value of *SDHB* RNA-seq quantification results. *P < 0.05; **P < 0.01; ***P < 0.001; ****P < 0.0001.

In order to clarify the significance of SDHB expression downregulation in ccRCC, we analyzed the correlation between SDHB expression level and clinicopathological characteristics. SDHB level gradually decreased as T stage, AJCC stage and Fuhrman grade progressed (**Figures 2A–E**). The low level of SDHB was also closely related with distant metastasis (**Figure 2F**). These results suggested that SDHB might be a potential prognostic marker for ccRCC patients.

We further observed the correlation between the low level of SDHB and the survival rates of ccRCC patients. The results showed that the low expression of SDHB was related with reduced overall survival (OS) and disease-free survival (DFS) time of ccRCC patients (**Figures 2G, H**), especially for patients in higher T stage, AJCC stage and Fuhrman grade (**Figures 2I–K**). Low level of SDHB was also closely related with relapse of ccRCC patients (**Figure 2L**). All these results revealed that low SDHB level is a potential prognostic marker for ccRCC patients.

### High Level of Methylation in *SDHB Gene Promoter* Leads to the Downregulation of SDHB Level in ccRCC Tissues and Correlates With the Malignant Degree of ccRCC Patients

**Figure 3:**
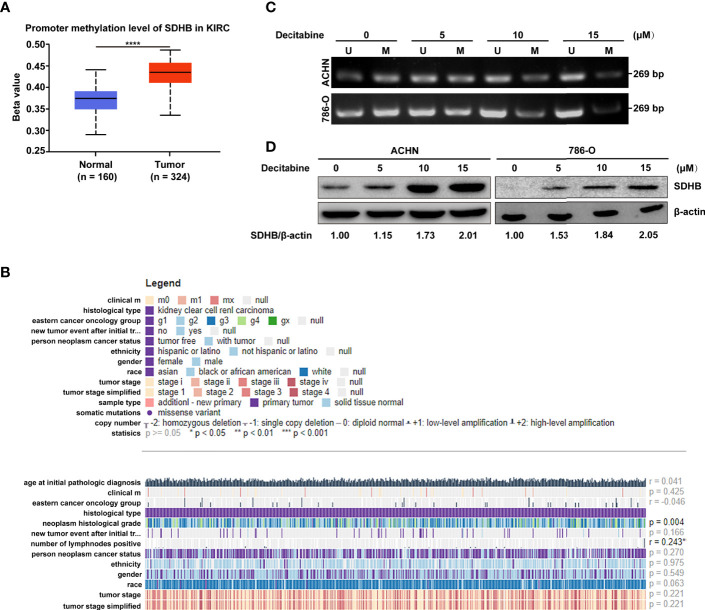
High methylation levels in ccRCC promote the downregulation of SDHB. **(A)** Methylation levels of *SDHB* gene promoter was increased in ccRCC tissues based on the TCGA_KIRC data. The Beta value indicated the level of DNA methylation, and the *P* value was derived from independent sample two tailed *t*-test. The data were presented as mean ± SD. ****P < 0.0001. **(B)** The correlation between SDHB methylation levels and clinical parameters in the MEXPRESS database. **(C)** MSP analysis of SDHB gene promoter methylation in ACHN and 786-O cells following treatment with increasing dose of methylation inhibitor decitabine. Bisulphite-modified DNA was amplified with primer pair specific for unmethylated (U) and methylated (M) alleles. The data are representative of three independent experiments. **(D)** Western blotting results showed that SDHB protein level was gradually increased in ACHN and 786-O cells following treatment with increasing dose of methylation inhibitor decitabine. β-actin was used as a loading control. The data are representative of three independent experiments.

Since the important role of SDHB downregulation in ccRCC tissues, we further explored the underlying mechanism of SDHB downregulation. The bioinformatics results showed that *SDHB* gene had low frequency in copy number variation or mutation for ccRCC (**Supplementary Figure 1**), but the methylation level of *SDHB* gene promoter in ccRCC tissues was significantly increased (**Figure 3A**). This reminded that the increase in the methylation level of *SDHB* gene promoter might be one of the mechanisms of SDHB downregulation in ccRCC. MEXPRESS database analysis result showed that the methylation levels of *SDHB* gene promoter in ccRCC tissues were significantly upregulated as grade progressed and positively correlated with lymph node metastasis (**Figure 3B**). This suggested the methylation in *SDHB* gene promoter had the important biological significance.

Hence, we further verified the causal relationship between methylation level of *SDHB* gene promoter and its expression level. We treated ACHN and 786-O cells with increasing concentrations of methylation inhibitor decitabine. As more methylation in *SDHB* gene promoter was inhibited, the methylation level of *SDHB* promoter gradually decreased (**Figure 3C**) and SDHB protein level was gradually increased (**Figure 3D**). This proved that the downregulation of SDHB in ccRCC is due to the increased methylation level in its promoter.

Due to the important biological significance of increased methylation levels in *SDHB* gene promoter, we explored its cause by analyzing expression levels of methylation-related writers and erasers. Results showed DNMT1, DNMT3A, DNMT3B and KDM1A were differentially expressed between ccRCC tissues and adjacent normal tissues (**Supplementary Figure 2A**). In addition, correlation analysis results showed that only demethylase KDM1A was correlated with *SDHB* promoter methylation (*r* = -0.1573, *P* < 0.05, **Supplementary Figure 2B**). These results suggest that KDM1A may regulate the methylation level of *SDHB* promoter in ccRCC.

### SDHB Suppresses the Proliferation and Migration of ccRCC cells

**Figure 4:**
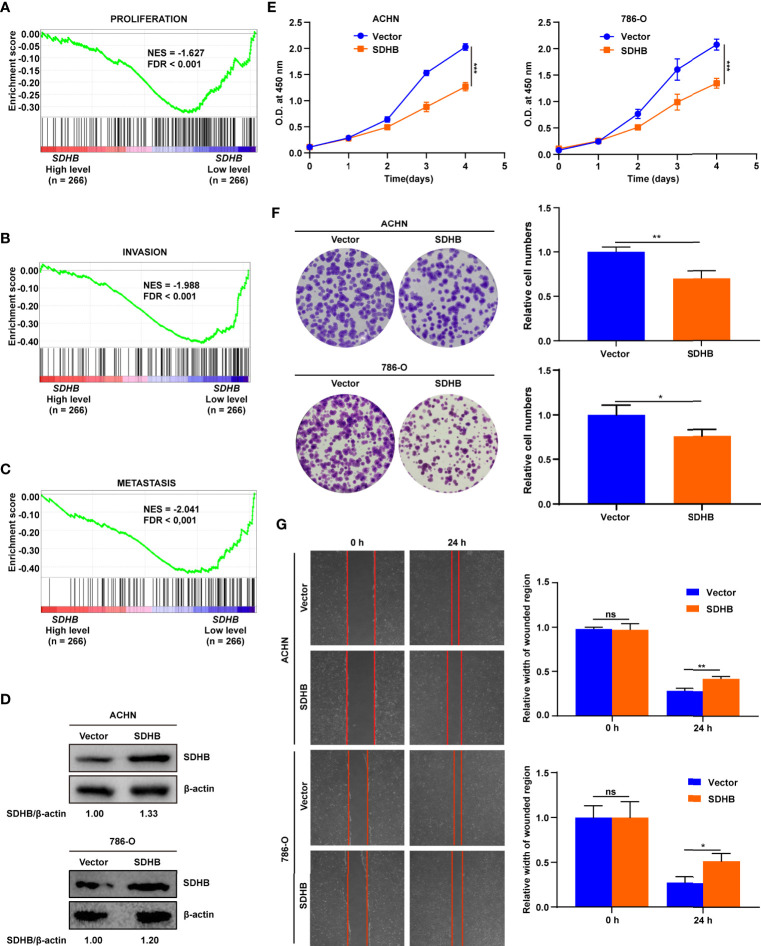
SDHB suppresses ccRCC cell proliferation and migration. **(A–C)** Enrichment plots of gene expression signature for proliferation (CHIANG_LIVER_ CANCER_SUBCLASS_PROLIFERATION_UP), invasion (PUIFFE_INVASION_ INHIBITED_BY_ASCITES_DN), metastasis (VANTVEER_BREAST_CANCER_ METASTASIS_DN) were obtained by GSEA according to *SDHB* mRNA levels. The ccRCC samples from TCGA_KIRC database were divided into high and low SDHB expression groups according to the median value of *SDHB* RNA-seq quantification results. **(D)** Western blotting results showed that SDHB was highly expressed in ccRCC cell lines following transfection with SDHB expression plasmid compared with control vector. β-actin was used as a loading control. **(E)** SDHB overexpression significantly suppressed ACHN and 786-O cell proliferation by CCK8 viability analysis. **(F)** SDHB overexpression significantly suppressed ACHN and 786-O cell colony formation by plate colony formation assay. **(G)** SDHB suppressed cell migration by wound healing assay. The relative migration distance is quantified. The data were presented as mean ± SD. *P* value was derived from repeated-measures ANOVA **(E)** and independent sample two tailed *t*-test **(F, G)**. **P < 0.01; ***P < 0.001. The data in **(D–G)** are representative of three independent experiments. *P < 0.05; ns, no significance.

The low expression of SDHB in ccRCC tissues also suggested that SDHB might be a tumor suppressor in ccRCC. In order to verify this kind of possibility, GSEA was performed based on the TCGA_KIRC dataset. The results showed that SDHB low expression was significantly correlated with ccRCC cell proliferation, invasion and metastasis (**Figures 4A–C**). This suggested that the low expression of SDHB may promote ccRCC tumorigenesis and development.

We used cell experiments to verify the tumor-suppressive effect of SDHB on ccRCC. SDHB was overexpressed in ACHN and 786-O cells (**Figure 4D**), and the overexpression of SDHB suppressed the proliferation, colony formation and migration of ACHN and 786-O cells (**Figures 4E–G**). This revealed that the low expression of SDHB promoted the occurrence and development of ccRCC *in vitro*.

### SDHB May Suppress ccRCC Tumorigenesis and Development by Inhibiting Glycolysis

**Figure 5:**
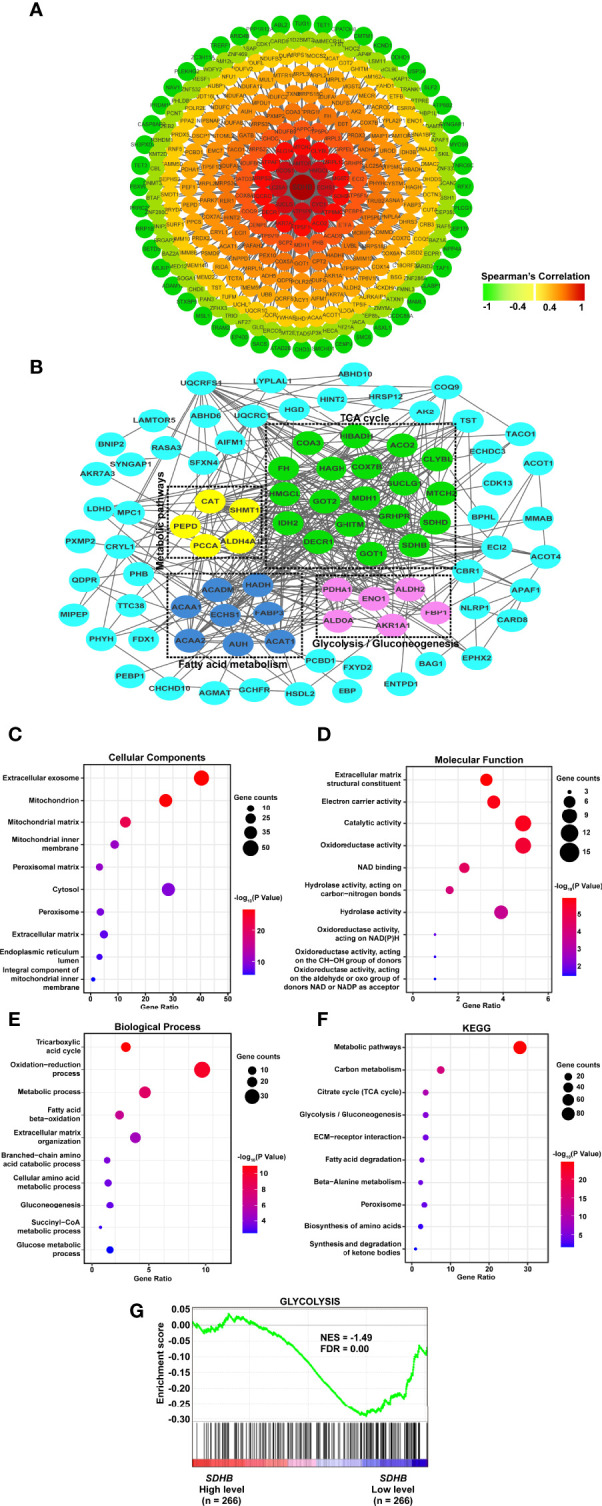
SDHB may suppress ccRCC tumorigenesis and development by inhibiting glycolysis. **(A)** Co-expression network of *SDHB* gene in ccRCC. Genes that were negatively correlated with SDHB were displayed in green, and genes that were positively correlated with SDHB were displayed in red. The darker the color, the stronger the correlation. **(B)** PPI network of SDHB-related DEGs. **(C–E)** Bubble chart according to GO (cellular component, molecular functions and biological processes) analyses of SDHB-related differentially expressed proteins. **(F)** KEGG pathway enrichment analysis of SDHB-related differentially expressed proteins. **(G)** Enrichment plot of gene expression signature for glycolysis (HALLMARK_GLYCOLYSIS). The ccRCC samples from TCGA_KIRC database were divided into high and low SDHB expression groups according to the median value of *SDHB* RNA-seq quantification results.

In order to clarify the mechanism by which SDHB suppresses the occurrence and development of ccRCC, we firstly analyzed the co-expressed genes of SDHB. Because co-expressed genes had the correlation with SDHB in expression level and these genes could suggest the function of SDHB in ccRCC. Co-expressed gene network was constructed and ATP5PB, HMGCL, LAMTOR5, AKR7A2, SUCLG1, ECHS1, MICOS10, CENPS, SCP2, ATP5F1C, SLC25A11, AKR1A1, ATP5MC3, *etc*. were moderately correlated with SDHB (**Figure 5A**).

Interaction occurred in all types of cells and were essential for the regulation of biological processes. If the genes which were differentially expressed between ccRCC and adjacent normal tissues, and correlated with SDHB could interact with each other, these genes would suggest the function of SDHB in ccRCC more clearly. Hence, interaction network was constructed by SDHB-related proteins based on the STRING database. A total of 113 genes were filtered into the target genes PPI network complex, containing 162 nodes, 388 edges (**Figure 5B**).

Then, we used SDHB-related proteins for GO and KEGG enrichment analyses. At the CC level, SDHB-related proteins were mainly enriched in exosomes and mitochondria. At the MF level, they were mainly enriched in extracellular matrix components, catalytic activity and redox activity. At the BP level, it was mainly concentrated in the TCA cycle, redox process and metabolic process (**Figures 5C–E**). KEGG pathway enrichment showed that SDHB-related proteins were mainly enriched in metabolic pathways, the TCA cycle and glycolysis pathways (**Figure 5F**).

We further analyzed the correlation between SDHB expression in ccRCC and glycolysis/TCA cycle through GSEA. the SDHB low expression was correlated with glycolysis (**Figure 5G**), but not the TCA cycle. In addition, we chose other metabolism-related proteins that interacted with SDHB and had a strong correlation with SDHB, analyzed the correlation between their expression levels and glycolysis by GSEA. The results showed the low expression of HMGCL and IDH2 were correlated with abnormal glycolysis (**Supplementary Figure 3**). All these results suggested that SDHB might suppress ccRCC tumorigenesis and development by inhibiting glycolysis.

### SDHB Overexpression Inhibits Glycolysis in ccRCC Cells

**Figure 6:**
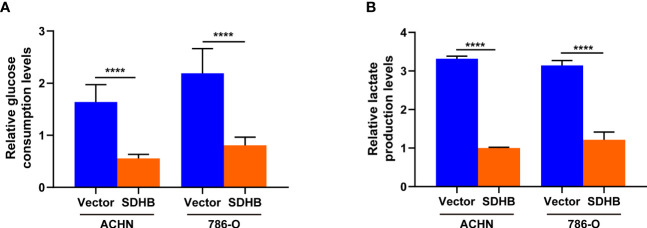
SDHB overexpression inhibits glycolysis in ccRCC cells. **(A)** Glucose level in SDHB-overexpressing ACHN and 786-O cells. **(B)** Intracellular lactic acid level in SDHB-overexpressing ACHN and 786-O cells. *P* value was derived from independent sample two tailed *t*-test. The data were presented as mean ± SD. ****P < 0.0001. The data are representative of three independent experiments.

Cancer cells promoted the rate of glycolysis by increasing glucose absorption (27). RCC was characterized by a higher rate of glycolysis and increased lactic acid production, of which glucose is the main energy source (28). Hence, we observed the glucose consumption and lactate production by SDHB overexpression. When SDHB was overexpressed in ccRCC cell line ACHN and 786-O, both glucose consumption and lactate production were inhibited (**Figures 6A, B**). This revealed that SDHB suppressed ccRCC tumorigenesis and progression by inhibiting glycolysis.

## Discussion

In this study, we found that SDHB expression was downregulated in ccRCC tissues and was gradually downregulated as malignancy progressed. SDHB could be a potential prognostic marker for ccRCC patients, especially for advanced ccRCC patients. SDHB suppressed ccRCC occurrence and development *in vitro* by inhibiting glycolysis. SDHB may serve as a potential therapeutic target for ccRCC patients.

The prognostic effect of SDHB was reported (29). However, they only analyzed the prognostic significance of SDHB in OS for 92 ccRCC patients. Based on this, we further analyzed the prognostic significance of SDHB in OS and DFS for 529 and 383 ccRCC patients from TCGA database, respectively. These results confirmed and broadened the prognostic value of SDHB due to the multicenter studies and the prognosis value for DFS. In addition, our IHC results for the specimen collected from the Affiliated Beijing Friendship Hospital, Capital Medical University also confirmed the potential prognostic value of SDHB in ccRCC. All these results verified the potential of SDHB as prognostic marker in ccRCC.

It was reported that SDHB was downregulated in colorectal cancer due to the upregulation of miR-142-5p (17). Our study revealed that the upregulation of methylation level for the *SDHB* gene promoter region may be the cause of SDHB downregulation in ccRCC. Unlike gene mutations, DNA methylation is a reversible process, so it is a promising target for drug development (30). Therefore, we speculated that the development of ccRCC could be regulated by targeting SDHB methylation level.

SDHB played an important role in the TCA cycle and oxidative phosphorylation. The loss or reduction of SDHB induced the conversion of mitochondrial respiration to cytoplasmic glycolysis (18). This metabolic change was related with tumor dedifferentiation, proliferation, migration and overall patient survival (18). Increased levels of aerobic glycolysis led to increased production of lactic acid. Lactic acid is a metabolite of aerobic glycolysis. It is transferred to the outside of the cell to form an acidic extracellular microenvironment (31–33), which was related with the increase in tumor aggressiveness (32, 33). Therefore, in some solid tumors, high lactate levels were associated with poor prognosis, high risk of metastasis and recurrence (33). The acidic extracellular microenvironment is not conducive to recognizing the immune effect of immune cells on cancer cells (10, 31) and led to the “immune escape” of cancer cells. Since aerobic glycolysis has a lower energy production efficiency than oxidative phosphorylation, cancer cells that rely on aerobic glycolysis need to consume more glucose to maintain energy balance (34). It makes cancer rob glucose from the surrounding environment. Due to the lack of glucose and other nutrients, many adjacent normal cells undergo apoptosis and necrosis, which in turn provides cancer cells with more living space. The dependence of cancer cells on aerobic glycolysis has been used as a target for anti-cancer therapy (35). ccRCC was characterized by enhanced glycolysis to maintain energy metabolism (36, 37). Our results proved that SDHB overexpression in ccRCC cells inhibited aerobic glycolysis. It is possible that ccRCC patients with poor prognosis suggested by low SDHB expression can be treated with anticancer drugs specifically targeting the Warburg effect.

In addition, SDHB was co-expressed with various metabolism-related genes in ccRCC. Lipid metabolism-related gene ECHS1 was reported by us to suppress ccRCC development and progression (38). HMGCL led to abnormal metabolism of ketone bodies in kidney cancer (39, 40), and SUCLG1 led to the coupling of succinyl-CoA hydrolysis with the synthesis of either ATP or GTP during the TCA cycle (40). Therefore, SDHB and these co-expressed proteins might synergistically cause abnormal metabolism of ccRCC, thereby promoting the occurrence and development of ccRCC. When we used SDHB-related DEGs to construct PPI network, SDHB is one of the hub genes for these co-expressed or related genes. This verified that SDHB indeed played the critical role in metabolism of ccRCC.

In summary, this study revealed that SDHB might predict the prognosis of ccRCC patients, especially advanced ccRCC patients. SDHB suppressed ccRCC occurrence and development *in vitro* by inhibiting aerobic glycolysis. These results help to provide new therapeutic target and prognostic marker for ccRCC.

## Data Availability Statement

Publicly available datasets were analyzed in this study. These data can be found here: https://www.ncbi.nlm.nih.gov/geo/query/acc.cgi?acc=GSE53757; https://www.cbioportal.org/study/clinical Data?id=kirc tcga.

## Author Contributions

ZF conceived the experiments, carried out all of the experiments and wrote the manuscript. JZ designed the experiment, verified the experimental data and wrote the manuscript. QS participated in the analysis of data. HY helped in verified the experimental data. All authors contributed to the article and approved the submitted version.

## Funding

This work was supported by grants from the National Natural Science Foundation of the People’s Republic of China (Nos. 81974415, 81600551), the Natural Science Foundation of Beijing (No. 7192021) and the Cultivation Fund Project of the National Natural Science Foundation in Beijing Children’s Hospital, Capital Medical University (No. GPMS202002)

## Conflict of Interest

The authors declare that the research was conducted in the absence of any commercial or financial relationships that could be construed as a potential conflict of interest.
