# Supplementary material for: SDHB Suppresses the Tumorigenesis and Development of ccRCC by Inhibiting Glycolysis
Source: Front Oncol. 2021 May 19;11:639408. doi: 10.3389/fonc.2021.639408 (PMC8170479; doi:10.3389/fonc.2021.639408)
Supplement: Supplementary file 1 [file DataSheet_1.docx]

**­
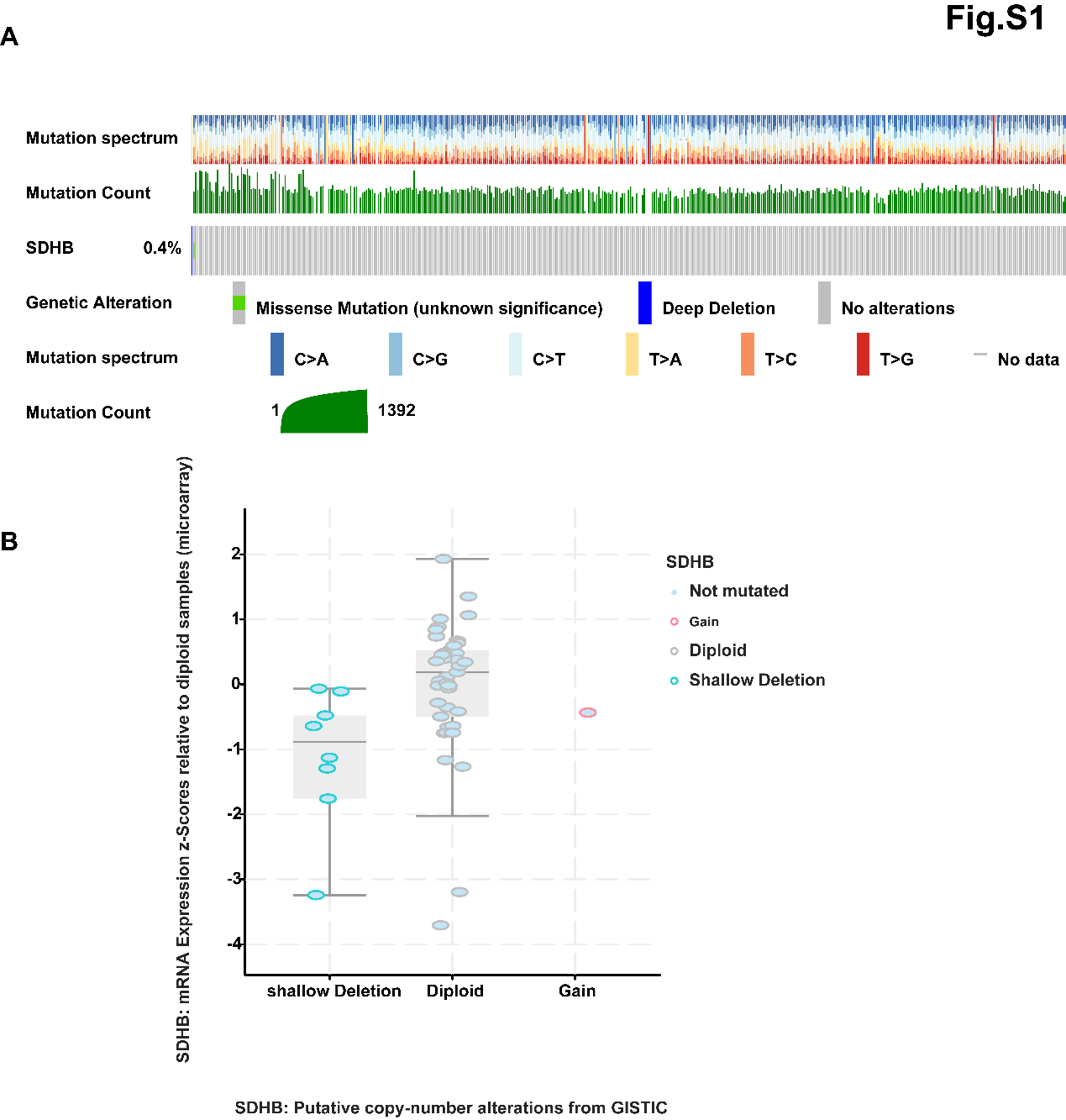
**

**Supplementary Fig. 1. *SDHB* gene has low frequency in copy number deletion and mutation.** (A) OncoPrint from cBioPortal showed that *SDHB* gene changed only in 0.4% of ccRCC cases. (B) With normal copy number samples (diploid) in contrast, samples with deleted copy number did not cause a significant decrease in *SDHB* mRNA levels.


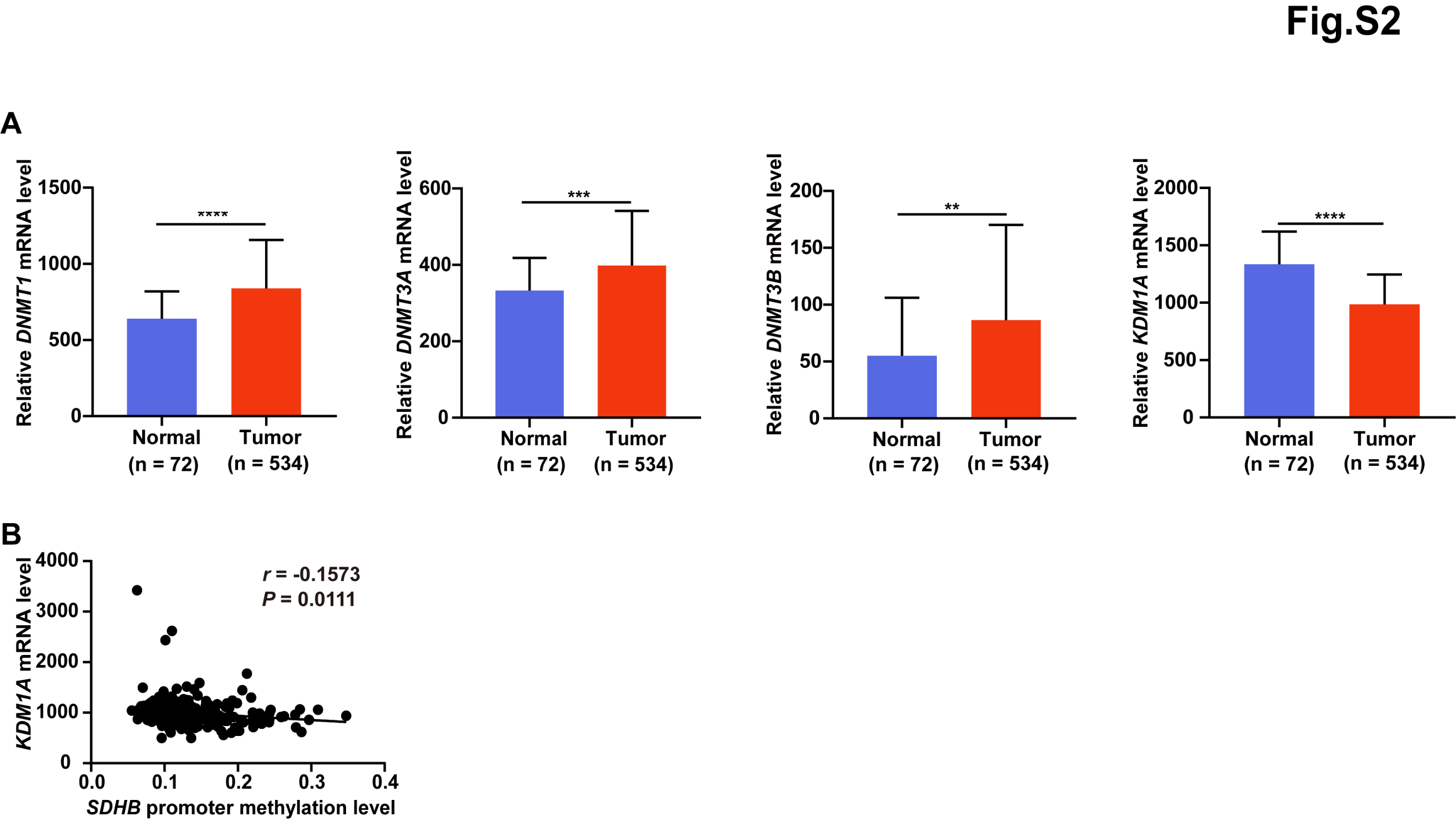


**Supplementary Fig. 2.** **KDM1A may be involved in the regulation of SDHB methylation levels in ccRCC.** (A) Expression level analyses of methylation-related writers and erasers showed *DNMT1*, *DNMT3A*, *DNMT3B* and *KDM1A* mRNA levels were differentially expressed between adjacent normal tissues and ccRCC tissues based on TCGA_KIRC data. (B) Based on TCGA data, the expression level correlation between methylation level in *SDHB* promoter and *KDM1A* mRNA level was analyzed.


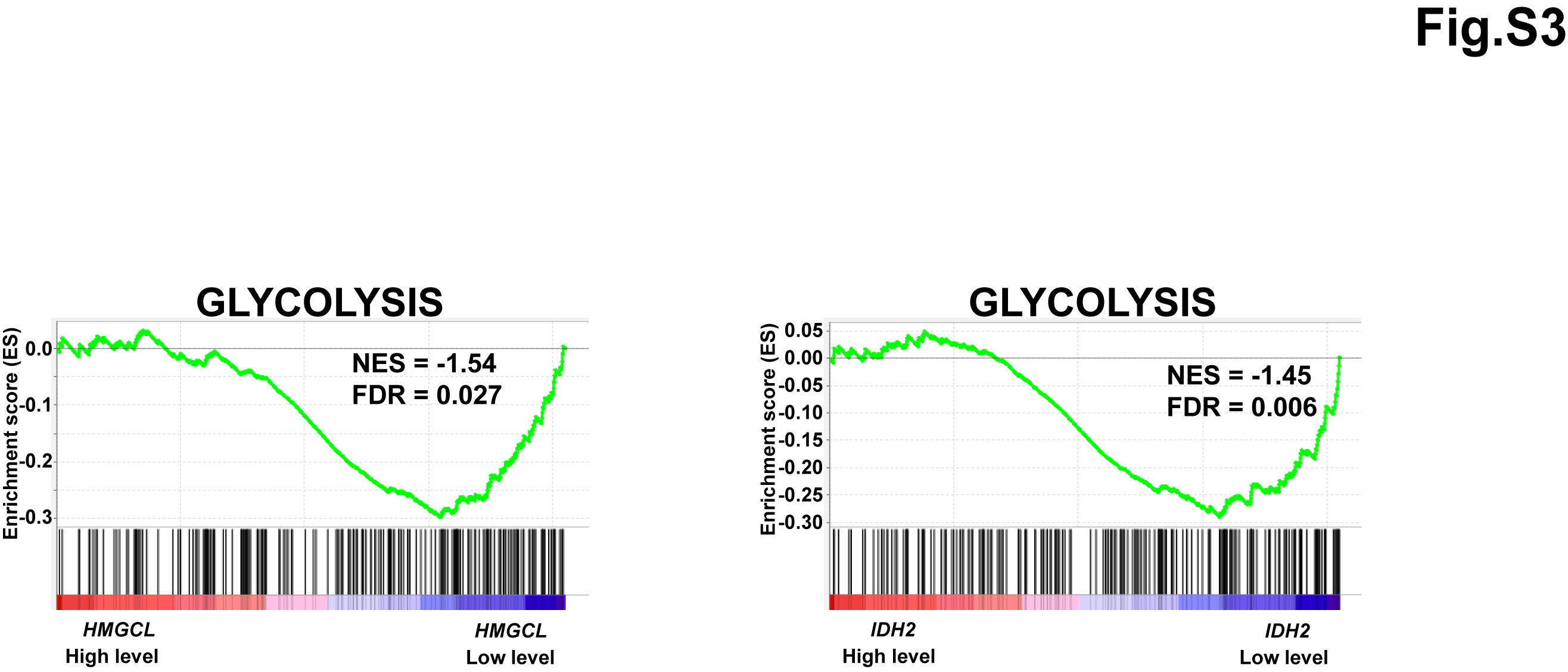


**Supplementary Fig. 3. Low level of HMGCL and IDH2 are correlated with abnormal glycolysis in ccRCC.** Enrichment plots of gene expression signature for glycolysis (HALLMARK_GLYCOLYSIS) were obtained by GSEA according to *HMGCL* and *IDH2* mRNA levels. The ccRCC samples from TCGA_KIRC database were divided into high and low gene expression groups according to the median value of gene RNA-seq quantification results.
